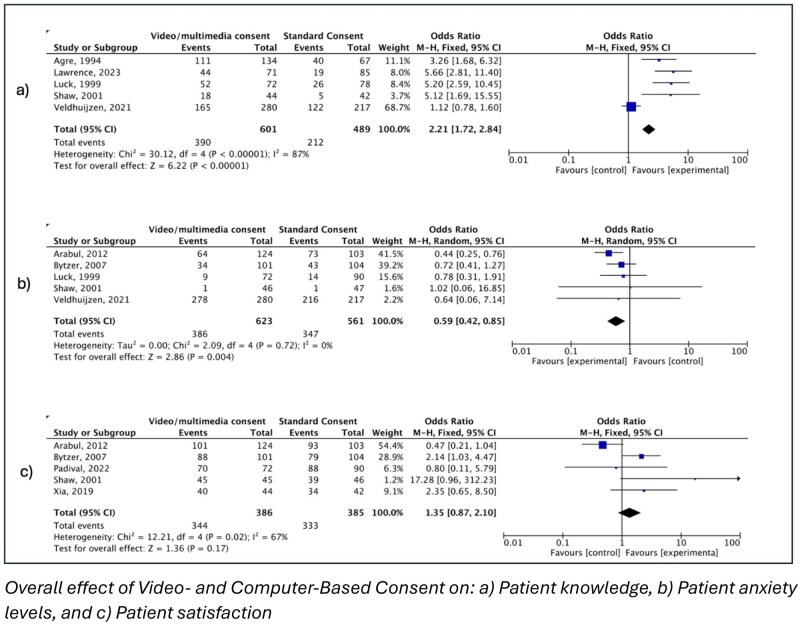# Poster Session I - A64 ENHANCING INFORMED CONSENT: A SYSTEMATIC REVIEW AND META-ANALYSIS OF VIDEO- AND COMPUTER-BASED APPROACHES COMPARED WITH CONVENTIONAL METHODS

**DOI:** 10.1093/jcag/gwaf042.064

**Published:** 2026-02-13

**Authors:** I Jamal, S Samnani, S Siyad, N S Rai, M Alkandari, A Albassam, A Chauhan, M Yaghoobi

**Affiliations:** Gastroenterology, McMaster University, Hamilton, ON, Canada; Gastroenterology, McMaster University, Hamilton, ON, Canada; Undergraduate Medicine, McMaster University Faculty of Health Sciences, Hamilton, ON, Canada; McMaster University, Hamilton, ON, Canada; Farwaniya Hospital, Farwaniya, Al Farwaniyah Governorate, Kuwait; Gastroenterology, McMaster University, Hamilton, ON, Canada; Gastroenterology, McMaster University, Hamilton, ON, Canada; Gastroenterology, McMaster University, Hamilton, ON, Canada

## Abstract

**Background:**

Informed consent is a critical component of patient-centered care. Traditional consent processes may not always optimize patient comprehension, satisfaction, or anxiety reduction, due to time constraints. Video- or computer-based consent interventions have emerged as potential tools to enhance these outcomes.

**Aims:**

To systematically review and synthesize evidence comparing video/computer-based consent processes with standard or conventional consent approaches, focusing on patient knowledge, satisfaction, and anxiety.

**Methods:**

A comprehensive search was conducted in MEDLINE, Embase, and the Cochrane Library. Eligible studies included randomized controlled trials and comparative studies evaluating video/computer-based versus conventional consent methods. Primary outcomes were patient knowledge, satisfaction, and anxiety. Risk of bias was assessed using standardized tools, and pooled analyses were performed where data permitted.

**Results:**

A total of 376 studies were identified through database searching, of which 9 studies met inclusion criteria. Compared with standard consent, video/computer-based interventions significantly improved patient knowledge (**OR 2.21, 95% CI 1.72-2.84, I^2^ = 87%**). Patient satisfaction was also higher with video/computer-based methods, but not significant (**OR 1.35, 95% CI 0.87-2.10, I^2^ = 67%**). Anxiety levels showed **significant difference/reduction** between groups (**OR 0.59, 95% CI 0.42–0.85, I^2^ = 0%**). The overall risk of bias was moderate, with frequent limitations in blinding and allocation concealment.

**Conclusions:**

Video- and computer-based consent processes enhance patient knowledge and satisfaction without increasing anxiety. These findings support incorporating digital tools as effective adjuncts to conventional consent, though further high-quality studies with standardized outcome measures are needed.

Overall effect of Video- and Computer-Based Consent on: a) Patient knowledge, b) Patient anxiety levels, and c) Patient satisfaction

**Funding Agencies:**

None